# Research on Monocular-Vision-Based Finger-Joint-Angle-Measurement System

**DOI:** 10.3390/s22197276

**Published:** 2022-09-26

**Authors:** Yongfei Feng, Mingwei Zhong, Fangyan Dong

**Affiliations:** Faculty of Mechanical Engineering & Mechanics, Ningbo University, Ningbo 315211, China

**Keywords:** monocular vision, human joint angle measurement, visual detection method, hand disability

## Abstract

The quantitative measurement of finger-joint range of motion plays an important role in assessing the level of hand disability and intervening in the treatment of patients. An industrial monocular-vision-based knuckle-joint-activity-measurement system is proposed with short measurement time and the simultaneous measurement of multiple joints. In terms of hardware, the system can adjust the light-irradiation angle and the light-irradiation intensity of the marker by actively adjusting the height of the light source to enhance the difference between the marker and the background and reduce the difficulty of segmenting the target marker and the background. In terms of algorithms, a combination of multiple-vision algorithms is used to compare the image-threshold segmentation and Hough outer- and inner linear detection as the knuckle-activity-range detection method of the system. To verify the accuracy of the visual-detection method, nine healthy volunteers were recruited for experimental validation, and the experimental results showed that the average angular deviation in the flexion/extension of the knuckle was 0.43° at the minimum and 0.59° at the maximum, and the average angular deviation in the adduction/abduction of the knuckle was 0.30° at the minimum and 0.81° at the maximum, which were all less than 1°. In the multi-angle velocimetry experiment, the time taken by the system was much less than that taken by the conventional method.

## 1. Introduction

The quantitative measurement of hand-joint range of motion (ROM) is important for clinicians to assess a patient’s level of hand disability and the effectiveness of intervention therapy. In the clinical setting, knuckle goniometers are often used to measure ROM due to their ease of use, portability, and affordability. However, these devices are time-consuming for single-joint angle measurements and do not allow simultaneous multi-joint angle measurements. Many experts and scholars have conducted in-depth research in the field of knuckle-angle measurement, including wearable-sensor-based knuckle-angle-measurement methods and vision-based knuckle-angle-measurement methods. Okuyama et al. developed a finger-joint-angle-measurement system based on flexible polymer sensors [1]. The system measures the flexion/extension movement of fingers by installing flexible polymer sensors on the surfaces of fingers, which can realize the detection of joint-angle changes during daily grasping movements. A three-dimensional (3-D) finger-motion-measurement system based on a soft sensor was proposed by Park et al. [2]. Changcheng et al. designed an integrated mechanical-sensor detection system, consisting of an angle-measurement device and a measurement circuit in order to achieve finger-joint measurement [3]. The effectiveness of the system was verified by joint-angle measurement, motion-law evaluation and object-grasping experiments, and the experimental results showed that the root mean square (RMS) of the DIP, PIP, and MCP angle-measurement errors were 0.36, 0.59, and 0.32 degrees, respectively [3]. It has been found that these wearable-sensor-based finger-joint-angle measurement methods have high accuracy in measuring finger joint angles, but the difficulty in wearing them has not been effectively solved in clinical applications for patients with hand motor dysfunction [4,5,6,7,8,9,10].

Vision-based knuckle-angle-measurement systems could realize the dynamic measurement of multi-joint angles without involving direct physical contact between the doctor and the patient’s hand. Vision-based measurement systems work by first capturing an image of the entire hand and then using computer-vision techniques to estimate the hand posture [11,12,13,14,15]. Commercial devices (such as Leap Motion) are currently used for hand-angle measurement [16,17] and, recently, they have been used in virtual-reality headsets (such as Facebook’s OculusQuest and Microsoft’s HoloLens2) equipped with hand tracking for human–computer interaction. The two main problems faced by current vision-based hand-posture estimation systems are the low accuracy of the knuckle-angle measurement and the high level of restriction on the camera view [18]. Lee J.W. et al. proposed a method of measuring finger-joint angles and finger forces in the process of maximum cylindrical grip using a multi-camera photogrammetric method with markers and a pressure-sensitive film, respectively [19]. The experimental results showed that this method can be used to judge the extension/flexion direction of the knuckle.

An industrial monocular-vision-based knuckle-angle-measurement system based on the existing computer-vision detection system is proposed in this paper [20]. This knuckle-angle-measurement system consists of a hardware system, a vision system, and a control system. The hand visual markers in the hardware system can simplify the difficulty of knuckle identification, and the use of high-resolution cameras can greatly improve the accuracy of the knuckle-angle detection. The active multi-angle light-detection system consisting of the control system, hardware system, and specified light source can adjust the light-irradiation angle and light-source-irradiation intensity to the marker by adjusting the height of the light source, thus enhancing the difference between the marker and the background, making the marker easy to the segment from the background and simplifying the marker-segmentation process.

## 2. Biological Structure of Human Fingers and Their Movement Characteristics

### 2.1. Structural Composition of the Human Hand

**Figure 1:**
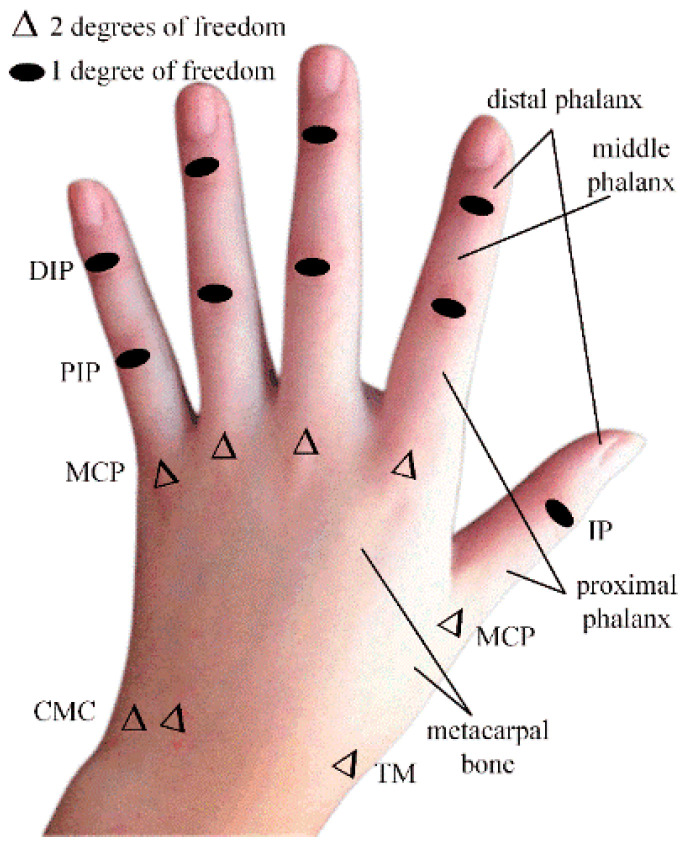
Structural components of the human hand.

The human hand consists of the index finger (IF), middle finger (MF), ring finger (RF), little finger (LF), and thumb (TUM). The IF, MF, RF, and LF consist of one degree of freedom (DOF) distal phalangeal (DIP), one DOF proximal phalangeal (PIP), and two-DOF metacarpophalangeal (MCP) and two-DOF carpometacarpal (CMC) joints, respectively. The thumb consists of a one-DOF distal phalangeal joint (IP), a two-DOF metacarpophalangeal joint (MCP), and a two-DOF carpometacarpal joint (TM) [21], as shown in Figure 1.

### 2.2. Finger-Movement Characteristics

**Figure 2:**
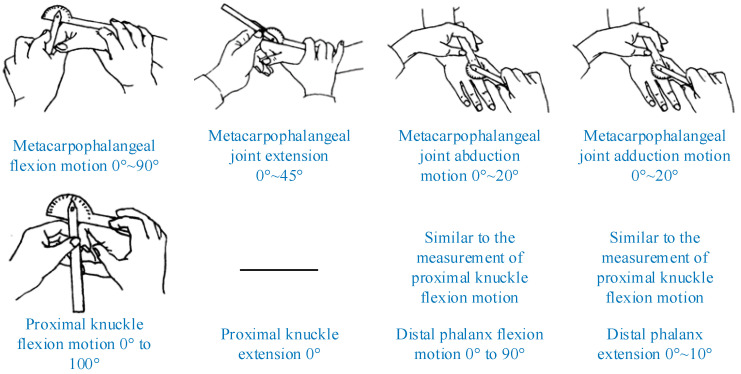
Human finger-joint range of motion and measurement methods.

The movement of hand joints is mainly manifested by the abduction/adduction and flexion/extension movements of the four fingers and the thumb. The movement of human fingers has the following characteristics: (1) the DIP and PIP joints of the four fingers other than the thumb are bound to each other and meet; (2) when the MCP joint of the four fingers other than the thumb is flexed, the adjacent MCP joint is also flexed. According to the Evaluation of Rehabilitation Therapy, the ROM of the human finger joint and traditional measurement methods can be determined, as shown in Figure 2.

## 3. Experimental-Platform Construction

Machine-vision technology has been developed, including hardware and software, but in the computer-vision measurement system, the design and layout of the lighting system is still a pivotal link, which can often significantly affect the performance of the vision-measurement system. A good illumination system can greatly enhance the difference between the measurement target and the measurement background, improve the system imaging, and make the target easier to identify and segment, thus simplifying the time and hardware cost required for program calculation. The different arrangements of light-source systems in the field of defect detection are often divided into passive multi-angle illumination-detection methods and active multi-angle illumination-detection methods. Considering the different characteristics of the two lighting methods, the active multi-angle lighting-detection method was selected as the light source arrangement method in the experimental platform.

### 3.1. Design of Experimental Platform

**Figure 3:**
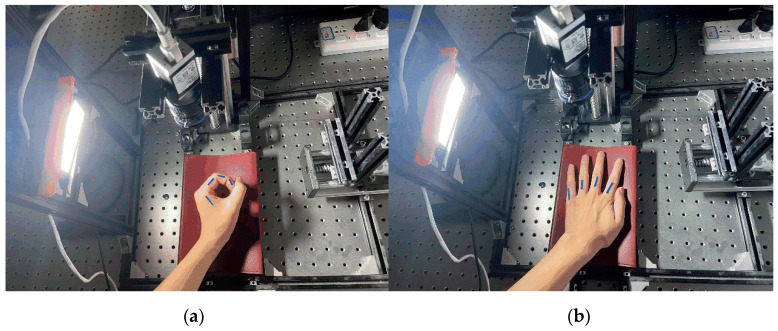
Finger-joint-angle-detection platform.

The core of the active multi-angle light-source detection method is the machine-vision-detection part; therefore, the quality of the acquired images and the speed of the image processing have a greater impact on the visual-detection effect. The quality of the camera hardware determines the quality of the image acquisition, and a high-performance, high-resolution camera can produce image data containing clear features under the irradiation of a highly stable light source, while a clear image is the basis for ensuring the stable operation of the image-processing algorithm and the detection effect of the system, which shows that the selection and design of the detection hardware are also particularly important. Based on the finger-joint-angle-measurement-system scheme, the actual system built in this study is shown in Figure 3. In Figure 3, Figure 3a represents the angle detection in the finger flexion/extension state, and Figure 3b represents the angle detection in the finger abduction/adduction state. Through this platform, high-quality multi-angle light-source-irradiated multivariate images can be acquired; subsequently, through the PC image-processing algorithm, these can be processed to segment the finger-joint identifiers in the image for the subsequent calculation of the finger-joint angle and length.

### 3.2. Light-Source Selection and Solution of the Single-Reflection Matrix

Industrial cameras are at the core of the vision-inspection system, and their main role is to convert the optical signal into an electrical signal and transmit it to the processing unit. As the most important part of the industrial camera, the light-sensitive element is of two main types: CCD (charge-coupled element) and CMOS (complementary metal oxide semiconductor). Furthermore, CCD technology is more widely used. Industrial cameras have many important parameters, such as resolution, shutter time, external trigger, frame rate, etc. Therefore, the vision-inspection system should take into account the needs of the inspection task to select the most appropriate camera. Depending on the interface type of the camera, it can be divided into USB, GigE, and camera link. Considering the advantages of the data-transmission speed, ease of use, and data-transmission distance, the GigE interface camera in Basler ace was selected.

In the inspection system, the choice of industrial lens directly affects the quality of the captured image. The industrial-lens parameters, such as interface type and CCD size, should be matched with the industrial camera. In addition, the aperture of the lens controls the light intake of the industrial camera, which exerts a direct impact on the brightness of the image; the focal length directly affects the size of the field of view, representing the vertical distance from the imaging plane to the center of the lens. Considering these lens characteristics, the lens selected in this study was TEC-V7X.

The light source is another important component of the visual inspection system, which is to determine the key to clear and stable imaging. The choice of the light source should highlight the object to be detected. According to the classification of light-emitting devices in the light source, the light source can be divided into fluorescent lamps, LED lamps, halogen lamps, etc., of which LED lamps are the most common. The light source selected for this paper was the ring light source of model R50-26-13, developed by Huakang Technology Company.

The transformation of the camera coordinate system, *x*-*y*-*z*, into the two-dimensional image coordinate system, *u*-*v*, is shown in Equation (1).

(1) $$\left[\begin{array}{l} u\\ v\\ 1 \end{array}\right]=s\left[\begin{array}{ccc} f_{x} & \gamma & u_{0}\\ 0 & f_{y} & v_{0}\\ 0 & 0 & 1 \end{array}\right]\left[\begin{array}{lll} r_{1} & r_{2} & t \end{array}\right]\left[\begin{array}{c} x_{W}\\ y_{W}\\ 1 \end{array}\right]$$

where $\left[\begin{array}{ccc} f_{x} & \gamma & u_{0}\\ 0 & f_{y} & v_{0}\\ 0 & 0 & 1 \end{array}\right]$ is the internal reference matrix of the camera and $\left[\begin{array}{lll} r_{1} & r_{2} & t \end{array}\right]$ is the external reference matrix of the camera. This leads to the formula for calculating the single-response matrix of the camera and the conversion formula for converting the pixel coordinates of the image to world coordinates as:

(2) $$\left\{\begin{array}{l} H=s\left[\begin{array}{ccc} f_{x} & \gamma & u_{0}\\ 0 & f_{y} & v_{0}\\ 0 & 0 & 1 \end{array}\right]\left[\begin{array}{lll} r_{1} & r_{2} & t \end{array}\right]=sM\left[\begin{array}{lll} r_{1} & r_{2} & t \end{array}\right]\\ sX=H^{-1}x \end{array}\right.$$

where ***H*** is the single-response matrix, ***x*** is the pixel coordinate in the image, and ***X*** is the world coordinate.

The above coordinate-system-conversion Equation (2) is used to obtain the single response matrix ***H*** from the pixel-coordinate system to one of the plane-coordinate systems (W) in space. Using ***H***, two points in the pixel-coordinate system can be converted into W. The distance s1 between two points in W is calculated, after which a ruler is used to directly measure the actual distance s2 between the corresponding two points in W. The error result of comparing s1 and s2 is 0.073 mm. However, when the relative distance between W and the camera changes, the error between s1 and s2 becomes dramatically larger. Therefore, during the finger-joint-angle measurement, the position of the detection plane relative to the camera should always be constant, and ***H*** should be updated in time when the distance of the camera relative to the detection plane changes.

## 4. Vision-Based Finger-Joint-Angle-and-Length-Detection Method

The finger-joint angle-and-length-detection method proposed in this paper is a joint-angle-detection method for visual-identifier-segmentation reprocessing. The method mainly consists of finger-joint-identifier pasting and image acquisition, visual identifier segmentation, the edge detection of visual identifiers, and joint-angle calculation based on the different joint identifiers of the finger. In the visual-identifier-segmentation method, the HSV color-space-conversion method and image-threshold segmentation method were adopted in this study to segment the finger-joint identifiers in the image. In the finger-joint-angle-calculation method, the inner and outer edge Hough straight-line-detection method and the least-squares method of fitting a straight line are used. Therefore, a finger-joint-angle image produces 2 × 2 joint angles and lengths, and the method that is ultimately closest to the real joint angle was selected as the finger-angle detection method for this paper by comparing the four joint angles with the real joint angle.

### 4.1. Vision-Based Finger-Joint-Angle-and-Length-Detection Method

**Figure 4:**
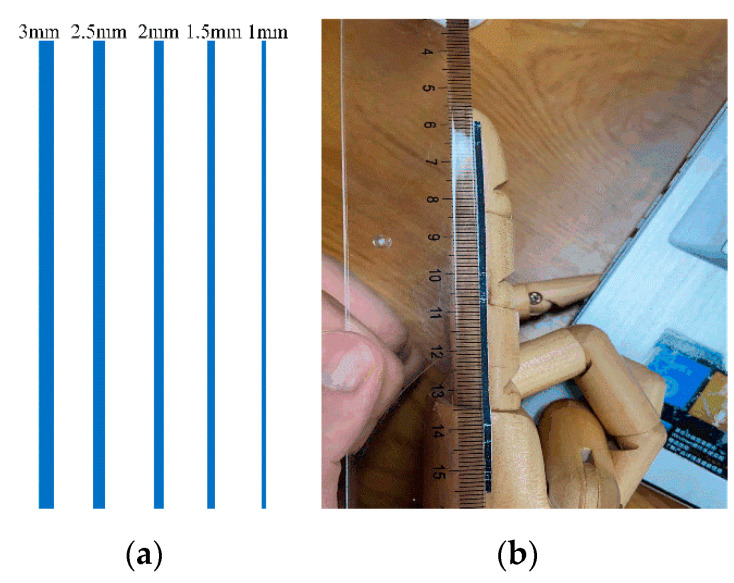
Finger-joint markers and their method of attachment.

When detecting the angle of each finger joint, firstly, the position of each finger bone in the image is identified and, secondly, the position and joint angle of each finger joint by the intersection point and the angle between each finger bone are identified. A finger-joint identifier for which it was easy to perform image segmentation was used for the identification of finger phalanges in the image. The finger-joint identifiers of different scales are shown in Figure 4a, and the most suitable finger-joint identifier was selected by comparing the accuracy of the angle detection of the identifiers at different scales. Figure 4b shows the method of attaching the finger-joint identifiers.

**Figure 5:**
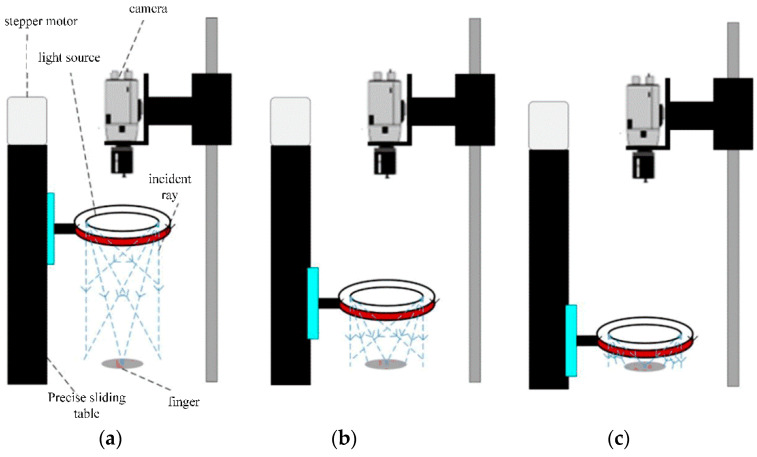
Image-acquisition method based on the active multi-angle light-source-detection method.

Since the light-source intensity and light-irradiation angle have a significant impact on the segmentation and extraction of finger-joint markers, the height of the light source can be adjusted to alter the light-irradiation angle and the light-source-irradiation intensity of the markers, enhancing the difference between the markers and the background, making it easy to segment the markers from the background and simplifying the marker-segmentation process. The image-acquisition method based on the active multi-angle light-source detection method is shown in Figure 5: (a) represents high angle lighting; (b) represents medium angle lighting; (c) represents low angle lighting.

### 4.2. Visual Marker Segmentation Methods

To obtain a better finger-joint-angle-detection algorithm, this paper uses the HSV color-space-conversion method and the image-threshold-segmentation method to extract the target finger-joint identifier in the image and different edge-detection algorithms to obtain the identifier edge coordinates and then calculates each finger-joint pinch angle by two different finger-joint-angle-detection algorithms.

(1) HSV color-space-marker-segmentation extraction with Canny edge detection

In HSV color space, H denotes color, S denotes shade when S = 0 only grayscale image, and V denotes light and dark, indicating the brightness of the color [22,23]. The conical model of HSV color space can be formed by erecting and flattening the central axis of the RGB-color-space 3D coordinates. The RGB–HSV color-space-conversion equations are shown in Equations (3)–(5).

(3) V=max(R,G,B)

(4) $$S=\left\{\begin{array}{cc} \frac{V-\min(R,G,B)}{V} & V\neq 0\\ 0 & \mathrm{other} \end{array}\right.$$

(5) $$H=\left\{\begin{array}{cc} 60(G-B)/(V-\min(R,G,B)) & V=R\\ 120+60(B-R)/(V-\min(R,G,B)) & V=G\\ 240+60(R-G)/(V-\min(R,G,B)) & V=B \end{array}\right.$$

**Figure 6:**
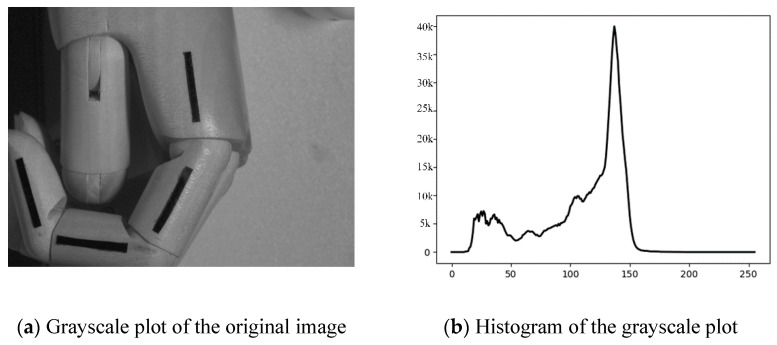
Grayscale conversion of the original image with the histogram.

In Equations (3)–(5), R, G, and B denote the three components of the three-dimensional coordinate axes in the RGB color space. The setting ranges of the three components of HSV are H: 100~130, S: 150~255, V: 130~255. The results of the specified color-region extraction are shown in Figure 6b. Canny edge detection is currently a commonly used edge-detection algorithm. It was proposed by John Canny in 1986 [23]. It is a multi-stage algorithm consisting of image-noise reduction, the computation of the image gradient, non-maximal value suppression, and threshold screening. Its formula for image-gradient calculation for edge detection is shown in Equation (6).

(6) $$\left\{\begin{array}{c} G=\sqrt{G_{x}^{2}+G_{y}^{2}}\\ \theta =\mathrm{atan}2\left(G_{y},G_{x}\right) \end{array}\right.$$

**Figure 7:**
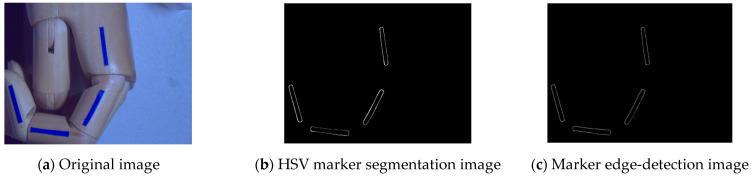
HSV marker segmentation and edge detection.

The *θ* in Equation (6) represents the gradient angle range of −π~π, which can be approximated as four angles, 0°, 45°, 90°, and 135°, representing the horizontal, vertical, and two diagonal directions, respectively. The Canny operator edge-extraction results are shown in Figure 7c.

(2) Image thresholding method with edge-contour extraction

The use of image segmentation to separate the target region from the background region can prevent the need to conduct a blind search on the image and greatly improve the processing efficiency of the image [24,25]. Threshold segmentation based on the grayscale histogram is simple to compute and is suitable for grayscale images where the target and background are distributed in different grayscale ranges, as shown in Figure 7 for the histogram of the original image.

**Figure 8:**
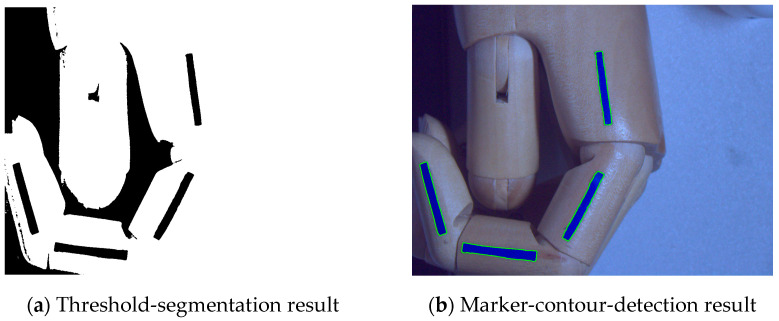
Image-thresholding segmentation and contour-detection results.

The image-segmentation formula based on different thresholds is shown in Equation (7), where *T* is the gray threshold; *f*(*x_i_*,*y_i_*) is the gray level of the detected image point, and A and $\bar{A}$ are the set gray level of the current position image. In this study, the gray level of the target image was set as 0, and the gray level of the other images was set as 255. The above operation was performed simultaneously by scanning the image by a line from two directions using a raster scan, which can prevent missing image information for various reasons, as shown in Figure 8a for the image after threshold segmentation. Next, the image contours were detected by the fine-contours function in OpenCV and, finally, the contours of the target identifier were filtered out automatically based on the similarity of the contour-enclosing area. The results of the target-identifier contour detection are shown in Figure 8b.

(7) $$g\left(x_{i},y_{i}\right)=\left\{\begin{array}{ll} \bar{A} & \mathrm{if}\;f\left(x_{i},y_{i}\right)>T\\ A & \mathrm{if}\;f\left(x_{i},y_{i}\right)⩽T \end{array}\right.$$

### 4.3. Joint-Angle-Calculation Method Based on Different Joint Identifiers of the Finger

(1) Hough straight-line detection method for inner and outer edges

The Hough transform was improved by Richard Duda in 1972. The method transforms a point in the data space into a curve in the ρ-θ parameter space so that points with the same reference-quantity characteristics intersect in the reference space after transformation. Subsequently, the detection of the characteristic straight line is completed by judging the accumulation degree at the intersection point. The expression formula of a straight line in the data space is shown in Equation (8), where *k* denotes the slope and *b* denotes the intercept.

(8) y=kx+b

The standard straight-line Hough transform uses the following parametric straight-line formula, as shown in Equation (9), where ρ is the perpendicular distance from the origin to the line and θ is the angle between ρ and the *x*-axis.

(9) xcosθ+ysinθ=ρ

**Figure 9:**
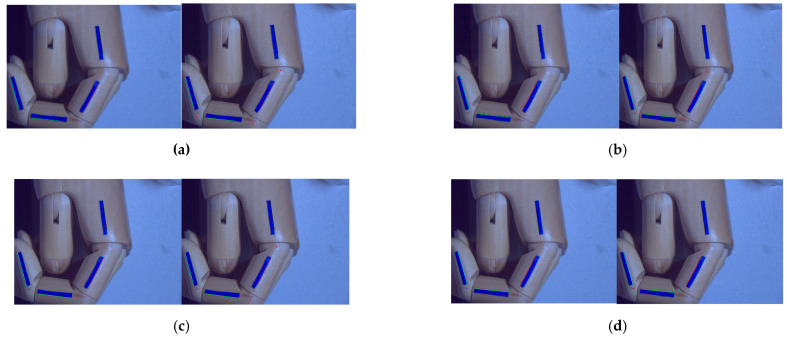
Inner and outer Hough straight-line detection results.

When different points on a straight line in the data space are transformed into a family of sinusoidal curves intersecting at *p* points in the parameter space, the detection of a straight line in the data space can be achieved by detecting the local maximum *p* points in the parameter space. The results of the detection of the inner and outer Hough straight lines for the target identifier are shown in Figure 9. Figure 9a represents the detection results of the Hough line on the outside of the HSV segmentation; Figure 9b represents the detection results of the Hough line inside the HSV segmentation; Figure 9c represents the detection results of the Hough line outside the threshold segmentation; and Figure 9d represents the detection results of the Hough line inside the threshold segmentation. The inner- and outer-edge Hough straight-line-detection method detects four straight lines on the inner edge and four straight lines on the outer edge of each identifier, after which the angle of each knuckle on the inner side of the identifier and the angle of each knuckle on the outer side are calculated using the finger-joint-angle-calculation method, and finally, the angle of each knuckle is found as $\theta_{i}=\frac{\theta_{iw}+\theta_{in}}{2}$ (*i* = 1, 2, 3).

(2) Least-squares fitting of the target identifier profile

The least-squares method was discovered by Legendre in the 19th century and takes the form shown in Equation (10). In Equation (10), $y_{i}$ is the observed value, i.e., multiple samples, and *y* is the theoretical value, i.e., the assumed fit function. $S_{\epsilon^{2}}$ is the objective function, i.e., the loss function, and the objective of the least-squares method is to model the fit function when the objective function is minimized.

(10) $$S_{\epsilon^{2}}=\sum {\left(y-y_{i}\right)}^{2}$$

**Figure 10:**
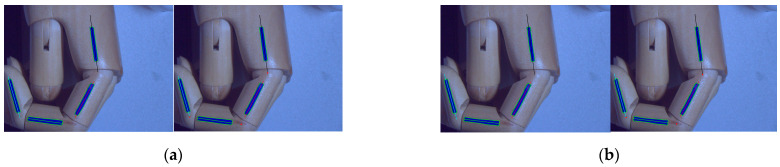
Least-squares linear-fit results.

To fit the four joint identifiers in the image as four straight lines, this paper assumes that the number of contour coordinates of each joint identifier is *n*. Assume that the equation of the straight line is *y* = *ax* + *b*, where *a* is the slope of the line and *b* is the intercept of the line. The least-squares method is used to solve for *a* and *b*, whose formulas are shown in Equation (11). The results of the least-squares method for fitting the straight line to the pixel points of the target identifier are shown in Figure 10. Figure 10a represents the line-fitting result of the HSV-segmentation least-squares method. Figure 10b represents the line-fitting result of the threshold-segmentation least-squares method.

(11) $$\left\{\begin{array}{c} b=\frac{\left(\sum_{i=1}^{N}x_{i}^{2}\right)\left(\sum_{i=1}^{N}y_{i}\right)-\left(\sum_{i=1}^{N}x_{i}\right)\left(\sum_{i=1}^{N}x_{i}y_{i}\right)}{N\left(\sum_{i=1}^{N}x_{i}^{2}\right)-{\left(\sum_{i=1}^{N}x_{i}\right)}^{2}}\\ a=\frac{N\left(\sum_{i=1}^{N}x_{i}y_{i}\right)-\left(\sum_{i=1}^{N}x_{i}\right)\left(\sum_{i=1}^{N}y_{i}\right)}{N\left(\sum_{i=1}^{N}x_{i}^{2}\right)-{\left(\sum_{i=1}^{N}x_{i}\right)}^{2}} \end{array}\right.$$

(3) Finger-joint-angle-calculation method

The relevant lines of finger-joint markers can be obtained by the above linear-detection methods. According to these lines, the head and tail coordinates of the four relevant lines of the four joint markers can be obtained, after which the angle between the joints of the fingers can be calculated by the formula of the angle between the two-dimensional vectors, as shown in Equation (12).

(12) $$\theta_{i}=\arccos\left(\frac{{\vec{a}}_{i}\cdot {\vec{b}}_{j}}{\left\|a_{i}\right\|\cdot \left\|a_{j}\right\|}\right)$$

In Equation (12), ${\vec{a}}_{i}$ and ${\vec{b}}_{j}$ are the vectors of two adjacent phalangeal identifiers and $\theta_{i}$ is the knuckle-joint angle. The finger-joint-angle measurements using different methods are shown in Table 1. The experiments showed better results with high-angle illumination. The results obtained for the detection of the human-hand model in the case of high-angle illumination are shown in the Table 1. HSV–HOISLM represents the HSV + Hough outer- and inner-straight-line method; HSV–LSFLKADM represents HSV + the method of least-squares-fitting linear-knuckle-angle detection; TS–HOMLDM represents the threshold segmentation + Hough outer medial linear-detection method; TS–LSFLM represents the threshold-segmentation + least-squares-fitting-line method; and TKAM represents traditional knuckle-angle measurement, as shown in Figure 2.

As can be seen from Table 1, the accuracy and reliability of the visual-based finger-joint-angle measurement method were demonstrated by comparing the measurement results of multiple visual-finger-joint-angle-measurement methods with those of the conventional finger-joint-angle measurement method, in which the angular deviation between the visual-based finger-joint-angle-measurement results and the conventional finger-joint-angle-measurement results were in the range of 0° to 2°. The maximum deviation in the comparison with the conventional knuckle-angle-measurement method was 2°, the knuckle where the maximum deviation was located was the DIP joint, and the visual-angle-measurement method that caused the maximum deviation was the HSV–LSFLKADM. The visual-angle-measurement method with the smallest mean value of the deviation of the finger-joint angle in comparison with the traditional finger-joint-angle measurement method was the TS–HOMLDM; therefore, this method was selected as the finger-joint-detection method for this paper.

## 5. Experimental Verification

In this study, nine healthy male volunteers aged between 20 and 25 were recruited for the experiment, and three different finger-joint angles were detected using the TS–HOMLDM for visual identifiers with widths of 1.5 mm, 2 mm, and 2.5 mm, respectively, to verify the monocular vision-based finger-joint-angle measurement system (MVBFJAMS) proposed in this paper to measure the accuracy of the test in comparison with the traditional inspection method and to determine the most appropriate visual identifier width. To ensure the reliability of the experiment, we invited professional physicians to measure different volunteer knuckle angles using the traditional method first, after which our group members measured different volunteer knuckle angles using MVBFJAMS. To verify the accuracy of the MVBFJAMS for finger-joint-angle measurement during finger extension/contraction, a control experiment was conducted using the conventional measurement method and the visual measurement method. This paper also verifies the speed of the knuckle detection by the visual inspection method by comparing the time used to detect and record 30 joint-angle data by the traditional method and the visual-inspection method. Table 2 shows the knuckle-joint-retention angles for different volunteers with different markers to verify the accuracy of the visual-detection method. The finger-bone-length data are not given because the actual joint position of the finger was uncertain.

The detection method in Figure 3a was adopted for the volunteers, and the detection results for the knuckle accuracy of the different volunteers at different scales of visual markers were obtained, as shown in Table 3.

**Figure 11:**
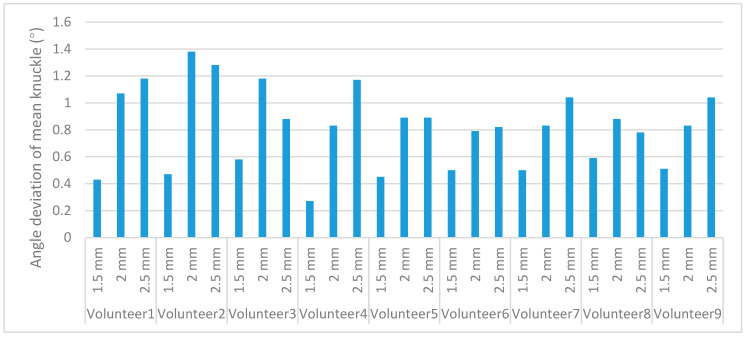
Deviation from the mean value of knuckle angle at different scales.

From Table 2 and Table 3, the deviations from the mean knuckle angle at different scale markers, shown in Figure 11, can be calculated.

As shown in Figure 11, the minimum-knuckle-angle mean deviation was 0.27° and the maximum-knuckle-angle mean deviation was 1.38° for the nine volunteers using visual identifiers at different scales. The knuckle-angle deviations for the nine volunteers using visual identifiers at a scale of 1.5 mm were 0.43°, 0.47°, 0.58°, 0.27°, 0.45°, 0.5°, 0.5°, 0.59°, and 0.51°, which were much smaller than the mean deviation of the knuckle angle when using other scales of visual identifiers. Therefore, the scale of a 1.5-millimeter visual marker was chosen as the test condition for the subsequent experiments. To verify the accuracy of the finger-abduction angle, three different finger-abduction-joint angles were measured using visual measures on nine volunteers, and the accuracy of the angles was verified using conventional methods. The results of the measurement of the three different abduction-joint angles are shown in Table 4.

In Table 4, Vmm represents the visual measurement method and Tmm represent the traditional measurement method. As shown in Table 4, the maximum and minimum knuckle-angle deviations of the nine volunteers were 0.81° and 0.30°, respectively. The mean values of the knuckles were 0.63°, 0.68°, 0.77°, 0.49°, 0.33°, 0.61°, 0.30°, 0.81°, and 0.83°, respectively. Table 5 shows the average time taken to measure and record the angle data of 30 joints for the 9 volunteers using the traditional method and the visual-detection method (including the time to paste the visual marker).

From Table 5, it can be seen that the time taken by the vision-based knuckle-angle-detection method is much less than that of the conventional knuckle-angle-detection method. This result was produced because the vision-based knuckle-angle-detection method not only enables the simultaneous measurement of multiple knuckles compared to the conventional knuckle-angle-detection method, but also increases the speed of the knuckle measurement and the speed at which the knuckle-angle data are recorded.

## 6. Conclusions

To solve the problems that the joint-angle measuring instrument takes more time to measure the angle of single joints in clinical medicine, and cannot measure the angles of multiple joints at the same time, a vision-based finger-joint-angle-measuring system was designed on the basis of the original visual-inspection system. The system consists of a hardware system, a control system, and a vision system. The active multi-angle-light-source-detection system composed of a control system and a hardware system can simplify the recognition process of visual markers by adjusting the height of the light source. The vision system is composed of an industrial camera and the knuckle-angle-detection method proposed in this paper. The knuckle-angle-detection method proposed in this paper is composed of finger-joint-marker pasting, image acquisition, visual-marker segmentation, visual-marker edge detection, and joint-angle calculation based on different finger-joint markers. In this study, each component of the method was analyzed and verified by experiments. These experiments proved that in the case of high angle illumination, the TS–HOMLDM should be adopted, and the visual marker with the scale of 1.5 mm was selected, since it had the highest measurement accuracy. The shortcomings of the current proposed MVBFJAMS are also very obvious. Firstly, the system requires a Basler ace camera, a TEC-V7X industrial lens, an R50-26-13 light source, and a computer, which makes it much more expensive than traditional knuckle-measurement methods and sensor-based methods; furthermore, the system can only achieve two-dimensional inspection at present.

The system is still in the experimental stage and has high requirements for the detection environment for light sources. Considering the complexity of the clinical environment, in order to improve the anti-interference capability of the system, we intend to add an opaque housing to the exterior of the device in the future in order to maintain the stability of the testing environment. In the next phase, we intend to add another depth camera to this system and fuse the texture information from the normal camera with the depth-camera depth information to build a model of the detector’s hand. Using this approach, three-dimensional detection can then be achieved to detect the angle of each finger joint of the hand. In the meantime, we will further validate the accuracy of the system through clinical trials, as well as the accuracy of the assessment of the level of handicap and the effectiveness of the intervention treatment.

## Figures and Tables

**Table 1:** Finger-joint angles measured by different methods.

	HSV + Hough Outer- and Inner-Straight-Line Method	HSV + Least-Squares-Fitting Linear-Knuckle-Angle-Detection Method	Threshold Segmentation + Hough Outer Medial Linear-Detection Method	Threshold-Segmentation + Least-Squares-Fitting-Line Method	Traditional Knuckle-Angle Measurement
MCP	145.02°	144.76°	144.95°	144.59°	145°
PIP	111.03°	109.38°	110.48°	111.26°	110°
DIP	111.83°	114.07°	112.09°	112.34°	112°
Length of proximal phalanx	26.94 mm	28.24 mm	27.37 mm	27.32 mm	27 mm
Length of middle phalanx	25.53 mm	25.53 mm	25.64 mm	25.26 mm	26 mm
Mean Angle deviation	0.407°	0.967°	0.207°	0.670°	

**Table 2:** Knuckle-retention angles under different markers in different volunteers.

	MCP (°)	PIP (°)	DIP (°)
Knuckle-hold angle under each marker	145	110	115
	160	130	110
	150	165	130

**Table 3:** Results of different volunteers’ visual-detection angles.

Volunteer	Mark on the Scale	MCP (°)	PIP (°)	DIP (°)	Length of Proximal Phalanx (mm)	Length of Middle Phalanx (mm)
volunteer 1	1.5 mm	144.72	109.31	115.42	45.52	30.23
		160.10	130.12	109.21	44.07	31.45
		149.48	165.72	130.31	45.31	30.21
	2 mm	145.21	109.10	114.42	46.21	31.03
		161.71	132.22	109.71	44.71	30.15
		151.31	167.28	130.02	45.49	29.24
	2.5 mm	144.72	108.91	115.92	46.71	29.02
		160.40	128.93	109.27.	43.93	30.51
		147.32	164.89	133.22	46.44	29.91.
volunteer 2	1.5 mm	145.31	110.21	114.71	47.22	33.47
		159.27	130.31	111.31	47.31	32.17
		150.32	164.44	139.74	46.28	31.95
	2 mm	143.31	110.72	116.71	46.93	33.36
		160.44	129.10	110.23	47.32	32.78
		150.77	165.69	131.21	48.91	34.19
	2.5 mm	146.21	110.79	114.49	48.31	35.66
		162.99	131.44	111.22	47.76	34.54
		150.55	167.21	131.59	47.77	31.22
volunteer 3	1.5 mm	144.81	110.47	115.69	43.17	27.49
		160.77	130.21	110.48	44.21	26.36
		150.06	165.56	131.81	42.89	28.91
	2 mm	144.31	111.81	114.01	44.33	29.36
		161.17	130.79	112.58	46.96	27.22
		150.97	163.84	130.91	45.89	26.54
	2.5 mm	146.79	110.11	115.98	43.22	27.77
		160.89	129.33	111.39	45.10	29.99
		150.34	166.79	130.44	45.78	26.53
volunteer 4	1.5 mm	145.32	110.17	114.87	45.17	30.24
		160.17	130.22	109.97	44.54	29.77
		150.27	164.90	131.07	45.98	29.31
	2 mm	146.32	110.54	115.94	43.33	27.45
		159.12	130.84	110.95	44.54	26.79
		149.71	165.55	128.77	42.59	26.34
	2.5 mm	146.71	110.21	116.19	43.24	28.79
		160.77	131.44	108.22	45.77	27.32
		151.45	165.99	130.97	43.35	28.23
volunteer 5	1.5 mm	145.21	109.55	115.94	40.22	23.33
		160.56	129.53	109.84	41.57	24.35
		150.41	165.77	129.92	43.98	23.47
	2 mm	145.99	109.21	116.31	42.22	25.22
		160.77	131.74	109.55	41.31	24.51
		150.22	166.33	130.44	39.45	23.91
	2.5 mm	145.97	109.22	115.33	40.58	25.33
		161.44	130.55	110.89	41.32	24.56
		148.97	165.33	131.75	43.77	22.22
volunteer 6	1.5 mm	145.31	109.12	114.33	40.12	30.21
		160.33	130.22	109.22	44.45	29.22
		150.22	165.72	130.33	43.43	27.34
	2 mm	145.33	110.47	115.33	39.65	28.79
		161.43	130.99	109.44	41.76	30.33
		150.67	165.33	131.65	42.22	30.67
	2.5 mm	146.12	110.22	115.48	45.97	30.15
		158.91	130.21	110.77	42.71	31.33
		149.23	163.47	131.22	42.45	29.78
volunteer 7	1.5 mm	145.33	110.32	116.12	36.45	27.13
		159.31	130.07	110.77	36.84	26.56
		150.21	165.22	129.22	37.32	26.32
	2 mm	146.71	110.42	113.41	34.78	25.72
		160.12	130.65	110.89	37.77	28.23
		151.14	164.31	130.22	37.96	27.45
	2.5 mm	145.42	110.31	114.21	39.03	29.81
		161.31	128.64	109.01	39.76	25.33
		152.12	166.21	129.13	38.78	25.91
volunteer 8	1.5 mm	145.32	110.77	114.57	43.15	27.49
		160.74	129.22	110.10	41.33	28.27
		151.12	165.33	129.38	44.54	27.39
	2 mm	145.72	110.31	116.66	42.56	29.72
		159.21	131.72	110.07	42.33	27.59
		150.56	166.77	130.33	41.12	28.23
	2.5 mm	145.32	110.07	115.21	44.45	30.02
		157.42	131.72	110.99	41.75	29.67
		150.22	166.23	129.25	45.39	28.37
volunteer 9	1.5 mm	145.31	110.23	115.76	35.46	23.57
		160.22	130.74	110.55	34.90	24.88
		159.31	165.21	130.90	37.04	24.42
	2 mm	146.13	111.31	114.31	36.24	25.56
		158.91	129.10	110.12	39.35	23.78
		149.01	165.12	131.14	37.67	24.33
	2.5 mm	143.21	109.22	115.33	38.91	24.89
		160.33	131.55	107.32	37.33	26.33
		151.33	165.77	129.22	37.57	23.91

**Table 4:** Measurement results of abduction/adduction knuckle angle.

Volunteer	Knuckle-Angle Measurement	Measuring Angle (°)			Mean Knuckle-Angle Deviation (°)
volunteer 1	Vmm	25.73	39.21	40.39	0.63
	Tmm	25	40	40	
volunteer 2	Vmm	24.32	39.03	40.41	0.68
	Tmm	25	40	40	
volunteer 3	Vmm	23.91	40.71	40.51	0.77
	Tmm	25	40	40	
volunteer 4	Vmm	24.41	39.93	40.33	0.33
	Tmm	25	40	40	
volunteer 5	Vmm	25.22	39.35	40.61	0.49
	Tmm	25	40	40	
volunteer 6	Vmm	24.12	38.77	41.12	0.61
	Tmm	25	40	40	
volunteer 7	Vmm	24.52	39.79	40.22	0.30
	Tmm	25	40	40	
volunteer 8	Vmm	24.91	38.54	40.89	0.81
	Tmm	25	40	40	
volunteer 9	Vmm	24.33	40.95	40.87	0.83
	Tmm	25	40	40	

**Table 5:** Time taken to measure and record data for 30 joint angles under different methods.

Method of Knuckle-Angle Detection	Time Taken to Measure and Record Knuckle Angles for 30 Times (s)
TMM	51.75
VMM	421.21

## Data Availability

Not applicable.
